# Right-Sided Dysplasia in Inflammatory Bowel Disease Is Not Associated with Conventional Risk Factors for Neoplasia

**DOI:** 10.3390/gastroent16020014

**Published:** 2025-04-07

**Authors:** Sumona Bhattacharya, William Beaty, Adam S. Faye, Jordan E. Axelrad

**Affiliations:** 1 Division of Gastroenterology, Department of Medicine, Grossman School of Medicine, New York University, New York, NY 10016, USA;; 2 Grossman School of Medicine, New York University, New York, NY 10016, USA

**Keywords:** inflammatory bowel disease, dysplasia, laterality, neoplasi1

## Abstract

**Introduction:**

In the general population, right I-sided dysplasia presents a higher risk for colorectal cancer (CRC) and metachronous dysplasia compared to left (L)-sided dysplasia. Given that patients with inflammatory bowel disease (IBD) are at higher risk for dysplasia than the general population, we sought to assess the risk factors as well as the differences in outcomes between patients with R-sided, L-sided, and both R- and L-sided dysplasia.

**Methods:**

A retrospective chart review was performed on patients at NYU Langone Health who had evidence of dysplasia on a colonoscopy between 2011 and 2021. Demographics and pertinent medical history were compiled. Cohorts were based on the dysplasia location (R-sided, L-sided, or R- and L-sided) and the IBD-related outcomes were analyzed.

**Results:**

A total of 71 patients had colonic dysplasia. The mean age was 54 years old (SD ± 17). The majority were male (72%), white (69%), and non-Hispanic (94%). A total of 76% had ulcerative colitis (UC) and 24% had Crohn’s disease (CD). Of all dysplastic lesions, 57 (80%) patients had unifocal disease and the remainder had multifocal disease. A total of 39 (55%) patients had R-sided dysplasia, 24 (34%) had L-sided dysplasia, and 8 (11%) had both R- and L-sided dysplasia. Patients with UC were more likely to have L-sided dysplasia (92% vs. 8% in CD; *p* = 0.04). Pseudopolyps were more likely associated with R- and L-sided dysplasia (38% in R- and L-sided dysplasia, 10% in R-sided dysplasia, and 4% in L-sided dysplasia; *p* = 0.03).

**Conclusions:**

Patients with UC had a higher risk for L-sided colonic dysplasia compared to patients with CD; however, there were no differences in the progression of dysplasia between those who had R-sided and those who had L-sided dysplasia. Larger studies are needed to assess the risk factors and outcomes related to the laterality of dysplasia and further validate these findings among patients with IBD.

## 1. Introduction

Inflammatory bowel diseases (IBD), comprising Crohn’s disease (CD) and ulcerative colitis (UC), are chronic inflammatory disorders of the gastrointestinal tract. Patients with UC and colonic CD are at an increased risk for colorectal neoplasia (CRN), which includes colorectal dysplasia and colorectal cancer.

The pathogenesis of CRN in patients with IBD includes colonic epithelial inflammation, mutations in genes, including the adenomatous polyposis coli (APC) gene, tumor protein 53 (p53), and microsite instability (MSI) [1]. Furthermore, active inflammation in IBD may produce proinflammatory cytokines which promote CRN. Multiple microRNAs (miRNAs) are associated with the development of CRN in patients with UC, for example, hsa-let-7d-5p and hsa-miR-331–3p [2]. The melanocortin system, particularly alpha-MSH, may protect against colonic inflammation [3].

Numerous risk factors have been identified for the development of CRN in patients with IBD. These include non-modifiable risk factors, such as male sex, personal history of CRN, family history of CRN (particularly significant if in first-degree relatives), and history of primary sclerosing cholangitis (PSC), as well as modifiable risk factors, such as smoking [1]. Intestinal inflammation is thought to be a key driver of CRN; therefore, patient-specific variables such as the disease duration, disease extent, and disease severity are also risk factors for CRN [1]. Finally, endoscopic complications demonstrative of severe and/or long-standing inflammation, including colonic strictures, a foreshortened tubular colon, and pseudopolyps, are also thereby associated with CRN, even if in an indirect fashion [1].

The laterality of dysplasia and the risk of progression to CRN, however, have not been evaluated in the IBD patient population. Studies on the non-IBD patient population have demonstrated notable and conflicting results. For example, a study by Qumseya et al. demonstrated that rigI(R)-sided polyps were more common than left (L)-sided polyps and more likely to be adenomas, but not necessarily more likely to be advanced adenomas or adenocarcinoma [4]. However, another study by Zare-Mirzaie et al. demonstrated a higher proportion of L-sided polyps with high-grade dysplasia (HGD) as compared to R-sided polyps [5]. Finally, a study by Fuccio et al. demonstrated that L-sided carcinomas have higher metachronous rates than R-sided carcinomas [6]. Given these findings in the non-IBD patient population, and the paucity of data evaluating this in patients with IBD, we sought to determine the risk factors as well as the differences in the natural history between R-sided and L-sided dysplasia in the IBD population.

## 2. Methods

An observational, retrospective chart review was performed on patients with IBD at NYU Langone Health, a tertiary academic center in New York City, New York, USA, who had evidence of dysplasia on a colonoscopy between 2011 and 2021. Patient demographics and IBD-related variables were recorded, including age, sex, race, ethnicity, smoking status (either current smoker, former smoker, never smoker), presence of PSC, IBD subtype (UC or CD), IBD extent by the Montreal classification [ulcerative proctitis (E1), L-sided colitis (E2), ulcerative pancolitis (E3), ileal CD (L1), colonic CD (L2), or ileocolonic CD (L3)] [7], duration of IBD, personal history of CRN, and family history of colorectal adenocarcinoma in a first-degree relative. Additional variables collected included the endoscopic and histologic severity of the disease as well associated complications of disease severity, such as the presence or absence of colonic strictures, pseudopolyps, and/or a foreshortened tubular colon.

At NYU Langone Health, high-definition white-light endoscopy without dye-spray chromoendoscopy is the standard for dysplasia screening, and all visible dysplasia is resected. During chart review, patients were categorized as either demonstrating unifocal dysplasia (which was defined as dysplasia present in only one location) or multifocal dysplasia (which was defined as dysplasia present in multiple locations). Further, dysplasia was categorized as either low-grade dysplasia (LGD) or HGD based on histopathology reports. Patients with histopathology reports of indefinite for dysplasia and adenocarcinoma were excluded from analysis in this study. Finally, cohorts were categorized based on the dysplasia location in the colon. Specifically, they were categorized as either R-sided (defined as located in the cecum, ascending colon, or transverse colon), L-sided (defined as located in the splenic flexure, descending colon, sigmoid colon, or rectum), or bilateral if multifocal dysplasia was present on both the R and L sides of the colon per these definitions.

Charts were reviewed to determine whether specific outcomes occurred after index colonoscopy with dysplasia. The outcomes noted included any dysplasia on subsequent examinations; this was characterized as either stable dysplasia, dysplasia progression, or dysplasia regression. Stable dysplasia was defined as either LGD on the index colonoscopy with LGD on the subsequent colonoscopy or HGD on the index colonoscopy and HGD on the subsequent colonoscopy. Dysplasia progression was defined as LGD on the index colonoscopy and either HGD or CRC on the subsequent colonoscopy, or HGD on the index colonoscopy and CRC on the subsequent colonoscopy. Dysplasia regression was defined as LGD on the index colonoscopy and no dysplasia on the subsequent colonoscopy, or HGD on the index colonoscopy and LGD or no dysplasia on the subsequent colonoscopy.

Secondary outcomes were captured and included if there was a need for future steroid use, biologic or small-molecule dose escalation, biologic or small-molecule changes in therapy, IBD-related hospitalization, surgery, or death.

Chi-squared and Fisher’s exact tests were performed to compare proportions between cohorts. The threshold for statistical significance was set at *p* < 0.05.

## 3. Results

Over the study period, we identified 71 patients with IBD and colonic dysplasia. At the time of dysplasia, the mean age was 54 years old (SD ± 17). The majority of patients with dysplasia were male (72%), white (69%), and non-Hispanic (94%). The mean BMI was 27, and most patients were never smokers (65%). A total of 76% of patients had UC, 24% of patients had CD, and a minority of patients had PSC (3%). The mean duration of IBD was 10 years. The majority of patients had no prior personal history of dysplasia (93%); 9% had a family history of CRN. Per endoscopy reports, no patients had strictures or a foreshortened colon; pseudopolyps were present in 11% of patients.

Of all dysplastic lesions, 57 (80%) patients had unifocal dysplasia and 20% had multifocal dysplasia. A total of 39 (55%) patients had R-sided dysplasia, 24 (34%) had L-sided dysplasia, and 8 (11%) had multifocal bilateral dysplasia (Table 1).

Patients with UC were more likely to have L-sided dysplasia (92% vs. 8% in CD; *p* = 0.04). Pseudopolyps were more often associated with multifocal bilateral dysplasia (38% in R- and L-sided dysplasia, 10% in R-sided dysplasia, and 4% in L-sided dysplasia; *p* = 0.03). Sex, smoking status, history of PSC, family history of CRC, endoscopic disease severity, and all outcomes studied, including dysplasia progression, were not associated with any differences between the cohorts based on laterality.

The outcomes studied were not statistically significant, including the need for surgery, steroids, biologic/small-molecule dose escalation or change, or hospitalization. No patients died in this cohort. None of the patients demonstrated the progression of dysplasia; however, 27% of the patients with R-sided dysplasia demonstrated dysplasia stability, 29% of the patients with L-sided dysplasia demonstrated dysplasia stability, and none of the patients with R- and L-sided dysplasia demonstrated dysplasia stability (*p* = 0.83). A total of 73% of the patients with R-sided dysplasia demonstrated dysplasia regression, 71% of the patients with L-sided dysplasia demonstrated dysplasia regression, and 100% of the patients with R- and L-sided dysplasia demonstrated dysplasia regression (*p* = 0.83).

## 4. Discussion

In this observational, retrospective, single-center study of IBD patients with colonic dysplasia, there were few clinical factors associated with dysplastic lesion laterality in the colon. L-sided dysplasia was associated with UC, whereas multifocal bilateral dysplasia was associated with the presence of pseudopolyps. We did not find any differences based on other covariates or in the clinically meaningful natural history outcomes between the three groups of patients stratified by dysplasia location, including the need for steroids, change in biologic or small-molecule therapy, IBD-related hospitalization, surgery, or dysplasia progression. These findings suggest that the laterality of the dysplasia in patients with IBD may not be associated with many of the conventional risk factors for dysplasia in the general population, and that it may not be associated with future disease activity outcomes.

The association between L-sided dysplasia and UC in this cohort was not unexpected given the characteristic distribution of UC. Inflammation in UC typically begins in the rectum and extends proximally in a continuous fashion to differing degrees in the colon. Subgroups of ulcerative colitis, according to the Montreal Classification, include ulcerative proctitis (E1), in which the distribution of inflammation present is limited to the rectum, L-sided ulcerative colitis (E2), in which the proximal distribution of inflammation present is distal to the splenic flexure, and extensive ulcerative colitis (also known as ulcerative pancolitis) (E3), in which the proximal distribution of inflammation is proximal to the splenic flexure (and often involves the entire colon) [7]. Of note, these phenotypes are not immutable, and UC patients’ distribution of inflammation may change over time to include additional proximal areas of the colon.

Given that all three of these subtypes of UC include the L-sided inflammation of the colon, albeit of varying proximal distributions, the higher likelihood of L-sided dysplasia demonstrated in this cohort is likely linked to chronic inflammation, a primary driver of CRN [1]. In IBD, recurrent and chronic inflammation of the colonic mucosa is believed to induce genetic and epigenetic changes associated with the development of colonic dysplasia and cancer. These changes include the promotion of mutations in the adenomatous polyposis coli (APC) gene, the promotion of microsatellite instability (MSI), and the promotion of the loss of tumor protein 53 (p53), among other critical changes associated with CRN propagation. In contrast, while CD may often involve the colon [7], the distribution of colonic inflammation may be patchy and classically spares areas of the left colon, such as the rectum; therefore, these patients may have a lower likelihood of L-sided dysplasia, as was seen in this cohort.

The left colon may derive benefit from improved surveillance, as compared to the right colon, thereby leading to the increased detection of L-sided dysplasia. One review from 2018 by Fischbach et al. analyzed 12 systemic reviews in the non-IBD patient population, including nine meta-analyses and 44 primary data records, to determine which variables impact the detection of precancerous lesions in the right colon [8]. The authors found evidence that the detection of precancerous adenomas in the right colon may be disproportionally negatively affected by variables such as shorter withdrawal times and inadequate bowel preparation as compared to the left colon [8]. Furthermore, Fischbach et al. found evidence that attachments to the colonoscope which serve to aid in the visual detection of precancerous lesions aid the right colon disproportionally more than the left colon, suggesting that the right colon is a more challenging area when it comes to the detection of precancerous growths, as compared to the left colon [8]. A recently published study by Baile-Maxía et al. in 2024 performed a multicenter, observational, retrospective analysis of post-colonoscopy colorectal cancer and determined that the majority of these lesions were found in the right colon; other variables associated with post-colonoscopy colorectal cancer included inadequate bowel preparation and piecemeal polypectomy [9]. Given the limited data collected in our study, we are not able to conclude whether any of the variables listed here affected the detection or surveillance of L-sided CRN in our cohort.

In our study, 10 patients with L-sided UC had R-sided dysplasia, in addition to 5 patients with ileal CD who also had R-sided dysplasia. As these patients’ IBD extent did not include areas of the bowel where CRN was detected, our findings may be attributable to the “field effect”, where normal tissue is susceptible to the development of neoplasia, which has been described in IBD [1,10]. The “field” in UC has been described as the entire colon, irrespective of the patient’s described phenotype [10]. It is not clear whether the field effect is applicable in CD between different segments of the bowel; however, if the same principles apply, it is possible that small bowel inflammatory disease affected the right colon. Other variables may also account for these findings. The IBD extent, while traditionally defined by the presence of endoscopic inflammation, has alternatively been defined by the presence of histologic inflammation [7]; therefore, it is possible that histologic areas of inflammation extended to areas which later developed CRN in our cohort, given that histologic inflammation is a key driver of CRN risk, as detailed above [1]. Other limitations to our study may also have contributed to these findings, including the retrospective study design (for example, patients may have been mislabeled with incorrect phenotypes or had other inaccurate documentation) and the nature of our academic center, where many patients seek consultation and often arrive with incomplete medical records. Therefore, these patient cohorts were included in our study.

Eleven percent of our patients had pseudopolyps noted on their endoscopic documentation. While our study demonstrated an association between multifocal bilateral dysplasia and pseudopolyps, the question of a direct or an indirect association between pseudopolyps and CRN itself is a controversial one. In one multicenter, retrospective cohort study by Mahmoud et al. published in 2019, the authors examined 462 patients with pseudopolyps of a total of 1582 patients (29.2% with pseudopolyps) [11]. The authors found that pseudopolyps were associated with more severe inflammation (adjusted odds radio (aOR): 1.32; 95% CI: 1.13–55), greater disease extent (aOR: 1.92; 95% CI: 1.34–2.74), and a lower likelihood of PSC (aOR: 0.38; 95% CI: 0.26–0.55). However, over a median follow-up of 4.8 years, Mahmoud et al. found no difference in the time to progression of advanced CRN, defined as either HGD or CRC, between those patients with pseudopolyps and those patients without pseudopolyps (*p* = 0.41). Furthermore, after adjusting for the cumulative inflammatory burden, they found no direct association between pseudopolyps and advanced CRN (adjusted hazard ratio: 1.17; 95% CI: 0.59–2.31). The authors of this study concluded that colonoscopy intervals should not be affected by the presence of pseudopolyps given their findings and should be driven by the cumulative inflammatory burden. However, Mahmoud et al. here only examined outcomes regarding the development of either HGD or CRC and did not include the development of LGD in their analysis.

A more recently published systematic review and meta-analysis from 2022 by He et al. examined 11 retrospective studies and 1 prospective study, including the Mahmoud et al. study [12]. Cumulatively, these studies included a total of 5819 patients, including 1281 patients with pseudopolyps (22% with pseudopolyps). The authors determined that patients with pseudopolyps did have an increased risk of CRN (odds ratio (OR): 2.01; 95% CI: 1.43–2.93) and even CRC (OR: 2.57; 95% CI: 1.69–3.91). Another meta-analysis by Shi et al. published in 2022 demonstrated similar results, with an increased risk of CRN in patients with pseudopolyps (36% with pseudopolyps; RR: 1.74; 95% CI: 1.35–2.24) [13], although none were adjusted for cumulative histologic or endoscopic inflammation exposure.

There are multiple mechanisms by which pseudopolyps could indirectly be associated with an increased risk of CRN over time given that pseudopolyps themselves are not thought to undergo direct malignant transformation [14]. One main mechanism for this is that pseudopolyps are thought to develop in areas of the colon affected by significant inflammation, as a part of the colonic epithelial regeneration process [14]. Specifically, pseudopolyps represent areas of the colonic mucosa that were spared by inflammation. Given that mucosal inflammation is the crucial driver of CRN [1], pseudopolyps could reasonably be indirectly associated with the development of CRN. Furthermore, the presence of significant pseudopolyposis can increase the difficulty of identifying true adenomatous polyps, potentially allowing CRN to progress to advanced stages prior to detection and resection. In patients with severe and extensive pseudopolyposis leading to technically challenging adenoma detection, colectomy should be considered [1].

He et al. similarly postulated that the associations found in their study between pseudopolyps, CRN, and CRC could be a result of prior severe inflammation or enhanced difficulty with the detection of neoplasia on an endoscopy [12]. While our study did not specifically examine the incidence of dysplasia in patients with pseudopolyps, the association that we found between these lesions and R- and L-sided dysplasia may be a reflection of the underlying inflammation that is posited to give rise to pseudopolyps [1,11].

None of our patients had strictures on their endoscopic documentation, possibly related to our small sample size; however, this is associated with CRN in the IBD population [1]. Similar to pseudopolyps, strictures are also associated with severe colonic inflammation. Strictures themselves may contain CRN or may indicate an overall increased disease severity that predisposes towards CRN in other areas of the colon.

Our findings stand in contrast to studies on the non-IBD population, particularly those that reported higher rates of progression of L-sided adenomatous polyps to HGD [5]. Although our sample was small, we had no patients with dysplasia progression from LGD to HGD on subsequent exams. Similarly, we did not find any association between dysplasia location and sex, in contrast to a large study in the non-IBD population showing higher odds of dysplasia in the male sex and similar data in the IBD population [4].

Our analysis did not demonstrate any association between the laterality of dysplasia and PSC (*p* = 0.18); however, our number of patients with PSC was small, representing just 3% of the sample size. Endoscopic and histologic inflammation is associated with the R colon in PSC [15] and, furthermore, DNA content abnormality is statistically more likely in the R colon [16].

Out study has multiple notable limitations. Our sample size was small, with only 24% having CD. The small sample size limited our ability to adequately detect the presence of dysplasia progression, stability, or regression, as well as long-term outcomes. Further, the demographics of our study were homogenous, as the vast majority of patients were male (72%), white (69%), and non-Hispanic (94%), meaning that the generalizability to other sexes, races, and ethnicities may be limited. Other limitations of our study include that it was performed at a single center and that it was retrospective in nature.

We note that our definitions of the R colon (defined as located in the cecum through the transverse colon) and L-sided (defined as located in the splenic flexure through the rectum) were assigned for the purposes of this study and may differ from those of other studies, as there are no generally accepted definitions for these terms. For example, Qumseya et al. defined the R colon as extending from the cecum to the splenic flexure [4], whereas Zare-Mirzaie et al. defined the R colon as extending from the cecum through the midpoint of the transverse colon, and the L colon as extending from the midpoint of the transverse colon through the rectum [5]. Furthermore, discrepancies between the endoscopic location and surgical location may exist [17], suggesting some degree of inaccuracy in the endoscopist’s assessment of the location of a particular lesion. The definitions used in our study sought to mirror the phenotypic definitions of UC [7], which was similar to other studies [6].

In summary, in this observational, retrospective study, the majority of clinical covariates and outcomes of dysplasia were not associated with the laterality of dysplasia, differing from data for the non-IBD population; however, our sample size was limited. We did find that patients with UC were more likely to have L-sided dysplasia, which may be linked with traditional UC phenotypes and improved endoscopic examination in the L colon. Additionally, pseudopolyps were more likely associated with R- and L-sided dysplasia, which may be linked with increased adenoma detection in patients with pseudopolyps. Larger studies and prospective studies are needed to further assess the risk factors and outcomes related to the laterality of dysplasia, as well as to validate our findings among patients with IBD. Such findings could aid in the risk stratification of patients, which may inform dysplasia screening intervals.

## Figures and Tables

**Table 1.** Overview of right-sided, left-sided, and right- and left-sided dysplasia among 71 patients at NYU Langone Health centers between 2011 and 2021.

	Right-Sided Dysplasia Only (*n* = 39)	Left-Sided Dysplasia Only (*n* = 24)	Right- and Left-Sided Dysplasia (*n* = 8)	*p*-Value
**Sex**				0.45
**Male**	26 (67%)	18 (75%)	7 (88%)	
**Female**	13 (33%)	6 (25%)	1 (13%)	
**Race**				0.30
**White**	27 (69%)	15 (63%)	7 (88%)	
**Black/African-American**	3 (8%)	6 (25%)	0	
**Asian/Pacific Islander**	4 (10%)	1 (4%)	1 (13%)	
**Other/Unknown**	5 (13%)	2 (8%)	0	
**Ethnicity**				0.18
**Hispanic**	1 (3%)	3 (13%)	0	
**Non-Hispanic**	37 (95%)	20 (83%)	8 (100%)	
**Unknown**	1 (3%)	1 (4%)	0	
**Smoking status**				0.13
**Active**	3 (8%)	1 (4%)	1 (13%)	
**Former**	7 (18%)	11 (46%)	2 (25%)	
**Never**	29 (74%)	12 (50%)	5 (63%)	
**PSC**	1 (3%)	0	1 (13%)	0.18
**Disease subtype**				0.04 *
**UC**	28 (72%)	22 (92%)	4 (50%)	
**CD**	11 (28%)	2 (8%)	4 (50%)	
**UC disease extent per Montreal classification** [7]				0.16
**Proctitis (E1)**	3 (13%)	6 (32%)	0	
**L-sided (E2)**	7 (29%)	8 (42%)	1 (25%)	
**Pancolitis (E3)**	14 (58%)	5 (26%)	3 (75%)	
**CD disease extent per Montreal classification** [7]				0.80
**Ileal (L1)**	3 (30%)	1 (50%)	1 (25%)	
**Colonic (L2)**	3 (30%)	0	2 (50%)	
**Ileocolonic (L3)**	4 (40%)	1 (50%)	1 (25%)	
**Mean duration of disease, years**	9	11	15	0.41
**Personal history of CRN**				0.04 *
**History of dysplasia**	0	2 (8%)	2 (25%)	
**History of CRC**	0	1 (4%)	0	
**Family history of CRC in first-degree relative**	4 (10%)	1 (4%)	1 (13%)	0.64
**UC endoscopic severity**				0.45
**MES 0**	9 (33%)	9 (41%)	0	
**MES 1**	7 (26%)	6 (27%)	1 (33%)	
**MES 2**	6 (22%)	6 (27%)	2 (67%)	
**MES 3**	5 (19%)	1 (5%)	0	
**CD endoscopic severity**				0.42
**SES-CD 0**	7 (100%)	1 (100%)	4 (80%)	
**SES-CD 4**	0	0	1 (20%)	
**Stricture**	0	0	0	NA
**Pseudopolyps**	4 (10%)	1 (4%)	3 (38%)	0.03 *
**Foreshortened colon**	0	0	0	NA
**Histologic severity**				0.93
**Normal/inactive**	17 (46%)	8 (36%)	4 (50%)	
**Mild**	5 (14%)	3 (14%)	2 (25%)	
**Moderate**	8 (22%)	6 (27%)	1 (13%)	
**Severe**	7 (19%)	5 (23%)	1 (13%)	
**Dysplasia**				
**Low-grade dysplasia**	36 (88%)	21 (92%)	7 (88%)	0.80
**High-grade dysplasia**	2 (5%)	2 (8%)	0	0.66
**Dysplasia focality**				NA
**Unifocal**	35 (90%)	22 (92%)	0	
**Multifocal**	4 (10%)	2 (8%)	100%	
**Dysplasia location**				NA
**Cecum**	8 (23%)	0	0	
**Ascending colon**	11 (31%)	0	0	
**Hepatic flexure**	3 (9%)	0	0	
**Transverse colon**	11 (31%)	0	0	
**Splenic flexure**	0	2 (8%)	0	
**Descending colon**	0	1 (4%)	0	
**Rectosigmoid colon**	2 (6%)	21 (88%)	0	
**Steroids**	4 (32%)	3 (23%)	2 (33%)	0.85
**Surgery**	1 (6%)	0	0	0.58
**Biologic/small-molecule dose escalation**	1 (6%)	0	0	0.58
**New biologic/small molecule**	4 (22%)	0	1 (17%)	0.20
**IBD-related hospitalization**	2 (11%)	0	1 (17%)	0.38
**Death**	0	0	0	NA
**Repeat endoscopic examination performed**	12 (67%)	8 (62%)	2 (33%)	NA
**Dysplasia progression**	0	0	0	NA
**Dysplasia stability**	3 (27%)	2 (29%)	0	0.83
**Dysplasia regression**	8 (73%)	5 (71%)	1 (100%)	0.83

* Met the threshold for statistical significance. PSC: primary sclerosing cholangitis; UC: ulcerative colitis; CD: Crohn’s disease; CRN: colorectal neoplasia; CRC: colorectal cancer; MES: Mayo endoscopic subscore; SES-CD: simple endoscopic score of Crohn’s disease; NA: not applicable; IBD: inflammatory bowel disease.

## Data Availability

Data is restricted due to privacy.
